# An efficient method for the immobilization of inulinase using new types of polymers containing epoxy groups

**DOI:** 10.1007/s10295-015-1619-4

**Published:** 2015-04-21

**Authors:** Mariusz Trytek, Jan Fiedurek, Beata Podkościelna, Barbara Gawdzik, Marcin Skowronek

**Affiliations:** Department of Industrial Microbiology, Institute of Microbiology and Biotechnology, Faculty of Biology and Biotechnology, Maria Curie-Skłodowska University, Akademicka St. 19, 20-033 Lublin, Poland; Department of Polymer Chemistry, Faculty of Chemistry, Maria Curie-Sklodowska University, Maria Curie-Skłodowska Sq. 5, 20-031 Lublin, Poland; Center for Interdisciplinary Research, The John Paul II Catholic University of Lublin, Konstantynów St. 1 F, 20-708 Lublin, Poland

**Keywords:** Copolymers, Epoxy groups, Eupergit, Immobilization, Inulinase

## Abstract

New glycidyl methacrylate copolymers containing different numbers of epoxy groups were synthesized and used to develop effective procedures for inulinase immobilization. The beneficial characteristics of the carriers included a high degree of crosslinking, stability at ambient temperature, an appropriate surface, and the presence of reactive epoxy groups. Some factors affecting the efficiency of immobilization of crude inulinase, including the kind and amount of carrier, the number of epoxy groups, as well as buffer pH and buffer concentration were examined. The yield of immobilization of this enzyme on the investigated type of microspheres was higher than on the commercial carrier, Eupergit^®^ C. After immobilization, the optimum temperature for inulinase activity shifted from 55 to 45 °C, whereas the optimum pH = 5 remained unchanged. The basic parameters of inulin hydrolysis were examined, and the possibility of applying the obtained biocatalyst in continuous conditions was tested. Inulin at a concentration of 0.5 % (w/v) was almost completely hydrolyzed to fructose (in a yield of 98 %) at a flow rate of 0.1 mL/min. A tenfold increase in the speed of flow resulted in an increase in the yield of oligosaccharides (DP2-DP6) up to ~41 % in the overall hydrolysate, as analysed by HPLC-RID and LC-ESI/MS. These results indicate that two forms of inulinase, an exo- and an endo-acting enzyme, were immobilized on our carrier. The enzyme showed good operational stability in a packed column over 28 days. There were no significant decreases in the efficiency of continuous hydrolysis during this time (about 17.4 % in comparison to its initial value).

## Introduction

Inulinases play an important role in the hydrolysis of inulin for commercial purposes. They are employed in the production of fructose syrups and fructooligosaccharides, which are extensively used in the pharmaceutical and food industries [23, 26]. Another application of inulinases is the production of ethanol from inulin [24, 45, 46].

Fungal inulinases are frequently composed of a mixture of fructanohydrolases with a high activity and stability [1]. The best known inulinases are those produced by species of *Penicillium* [25], *Aspergillus* [5], and *Kluyveromyces* [10].

Inulinases with a high thermostability have been obtained from strains of *Aspergillus* spp. and thermophilic bacteria. Molecular cloning of inulinase genes from different sources has revealed that beside conserved domains, endo- and exo-acting inulinases show motifs which are distinct for the two classes of enzymes [34].

While free enzymes can be used efficiently in batch-type processes, they do not lend themselves to use in continuous, industrial-scale processes. Immobilization enables repetitive use of enzymes and hence significant cost savings. From the technological point of view, immobilized enzymes can easily be separated from the reaction liquid and make laborious separation steps unnecessary. Additional benefits arise from stabilization against harsh reaction conditions which are deleterious to soluble enzyme preparations [40]. The immobilized enzymes may be employed in various reactor systems, such as in packed columns and stirred tank reactors, depending on the nature of the substrate, which is being biocatalytically reacted [45].

Methods proposed for immobilization of an enzyme are based on the use of a carrier in the form of a solid support made from inorganic or organic material. Such materials include gamma-alumina, titania, activated granular carbon, granular diatomaceous earth, glass beads, porous glass, pumice-stone, silica gel, metal oxide and aluminum oxide. For example, inulinase produced by the yeast strain *Kluyveromyces marxianus* var. *bulgaricus* has been experimentally immobilized on various materials, such as activated carbon, diatomite, hen egg shell, Amberlite, porous silica and gelatin [28].

A compound, or a mixture of compounds, is used to attach the enzyme to a carrier, with polyethylenimine and glutaraldehyde in particular being cited. However, such methods can be disadvantageous in that the enzyme is not tightly held (by either being bonded thereto or being entrapped therein) to the carrier. Thus, the enzyme can become “detached” (unbonded) from the carrier becoming “free” in the reaction medium. In fact, the forces which exist between the enzyme and the carrier so as to hold them together are often quite weak, such that the enzyme is readily desorbed from the carrier in the presence of the substrate being processed, and lost in the reaction medium [7, 9, 28, 42, 44, 45]. Due to these circumstances, introduction of appropriate substituents/groups that are capable of enhancing the anchoring of enzymes within carriers is desirable. Epoxy groups are known for their high affinity for proteins; however, too many bonds between an enzyme and a carrier may change the spatial structure of the enzyme and, in consequence, decrease its activity [12].

In a previous study [36], a mutant of 20 OSM with an extracellular inulinase activity about twofold higher than that of the wild strain was obtained after mutagenic activation of *A. niger*. The mutant, immobilized on polyurethane foam, was employed for enhanced production of free inulinase in a bioreactor with unconventional culture oxygenation (using H_2_O_2_) [39]. The aim of this present study was to develop an effective procedure for the immobilization of inulinase from *A. niger* using new types of polymers containing epoxy groups and to optimize some parameters of this process. The possibility of applying the obtained biocatalysts in continuous hydrolysis conditions was also tested.

## Materials and methods

### Chemicals

Glycidyl methacrylate (GMA), decan-1-ol and *bis*(2-ethylhexyl)sulfosuccinate sodium salt (DAC, BP) were purchased from Fluka AG (Buchs, Switzerland). α,α′-Azoiso-*bis*-butyronitrile (AIBN), 1,4-divinylbenzene (DVB) and styrene (ST) were obtained from Merck (Darmstadt, Germany). Reagent grade acetone, methanol, chlorobenzene, chloroform, acetonitrile, hexane, toluene and tetrahydrofurane (THF) were from POCh (Gliwice, Poland). *Bis*[4(2-hydroxy-3-methacryloyloxypropoxy)phenyl]sulfide (BES.DM) was obtained by a procedure described in our previous work [30].

### Synthesis of polymers

Polymers containing epoxy groups were obtained in the form of microspheres in a suspension–emulsion copolymerization procedure [29, 31]. The experimental parameters of the syntheses are presented in Table 1.

**Table 1:** Experimental data and characterization of the obtained microspheres

Ratio of monomers (% mol.)	BES.DM	GMA	ST	DVB	AIBN	*L*_EP_ (mmol/g)	
	(g)					Theo.	Det.
1:1 (DVB:GMA)	0	10	0	9.15	0.191	3.67	1.71
1:1 (BES.DM:GMA)	10	2.83	0	0	0.128	1.56	1.40
1:6 (BES.DM:GMA)	10	16.97	0	0	0.270	4.44	2.79
1:1:3 (BES.DM:GMA:ST)	10	2.83	6.39	0	0.192	1.05	0.29

**Fig. 1:**
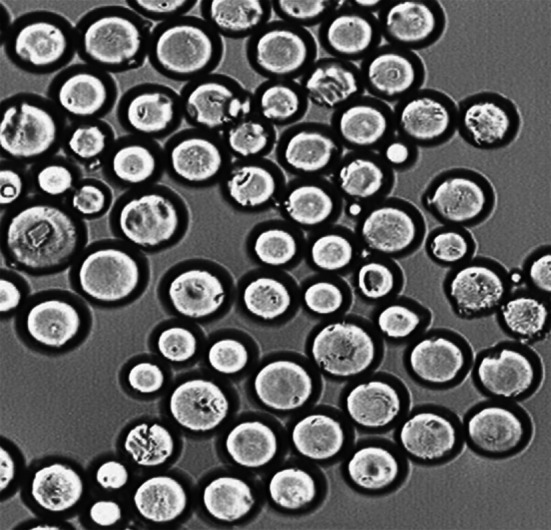
Image of BES.DM-GMA microspheres (in acetone) used for immobilization of inulinase

The dispersion medium was prepared by dissolving 1 wt% *bis*(2-ethylhexyl)sulfosuccinate sodium salt (DAC, BP) in deionized water. The synthesis was carried out in a three-necked flask equipped with a stirrer, a water condenser and a thermometer. The initiator AIBN (1 wt%) was dissolved in monomers, and then the mixture was diluted with a mixed solvent (toluene/decan-1-ol) taken in 1/1 (w/w) ratio. The reaction mixture was stirred at 350 rpm for 18 h at 80 °C. The copolymers obtained were washed with distilled water, filtered off, dried and extracted (in acetone and methanol) in a Soxhlet apparatus. The polymerization conditions applied yielded about 80 % of beads in the size range of 10–30 μm (Fig. 1).

### Characterization of the polymers

Chemical structures of the newly obtained copolymers were confirmed by Fourier transform infrared (FTIR). Spectra were recorded on a Perkin-Elmer 1725 X spectrophotometer in the 400–4000 cm^−1^ wavenumber range using KBr pallets.

The beads were examined using an atomic force microscope AFM by Nanoscope V (Veeco–Bruker, USA) operating in the contact mode.

The HCl/dioxane method was used to determine the number of epoxy groups (*L*_EP_). The assay involved a quantitative reaction of HCl with epoxy groups in the environment of dioxane followed by titration of the remaining hydrogen chloride in an ethanolic solution of NaOH. The epoxide content was calculated from the difference between the blank and the test sample after titration.

The porous structure of the copolymers was investigated by nitrogen adsorption at 77 K using an adsorption analyzer ASAP 2405 (Micrometrics Inc., USA). The specific surface areas were calculated by the BET method assuming that the area of a single nitrogen molecule in the adsorbed state is 16.2 Å^2^. Pore volumes and pore size distributions were determined by the BJH method.

### Recovery of crude inulinase

Inulinase was synthesized in a submerged culture of *A. niger* 20 OSM as reported previously [37]. The fermented broth was centrifuged (2500×*g*, 10 min, 4 °C) in a refrigerated centrifuge (6K15—Sigma Laboratory Centrifuges, Osterode, Germany), and the supernatant was concentrated fourfold in a R-205 rotary vacuum evaporator (Büchi Labortechnik AG, Switzerland).

### Immobilization of inulinase

Concentrated post-culture liquid (1 mL containing 200 units of inulinase) and an appropriate amount of polymer microspheres (0.2–1.0 g) were added to 4 mL of phosphate buffer (0.75 M, pH 6.5, unless stated otherwise). The mixture was incubated at 30 °C under gentle stirring for 24 h. After incubation, the carrier-bound enzyme was recovered by filtration, washed with 250 mL of distilled water and kept in water at 4 °C until further use.

### Inulinase assay

For the immobilized enzyme assay, a reaction mixture containing an appropriate amount of immobilized inulinase and 10 mL of 0.5 % inulin (from Dahlia tubers, Sigma Chemical Co., St Louis, MO, USA) dissolved in 0.1 M acetate buffer (pH 5.0) was incubated on a rotary shaker at 40 °C. After 20 min of incubation, the increase in reducing sugars was estimated with the 3.5-dinitrosalicylic acid method [22]. Absorbance was measured at 550 nm. As a control, an adequate volume of free enzyme was used in place of the immobilized enzyme.

One unit (U) of inulinase activity was defined as the amount of the enzyme which produced 1 μmole of reducing sugars per min under the above conditions.

The immobilization yield was calculated as the ratio of the total activity of immobilized inulinase to the total activity of the free enzyme used for immobilization.

### Determination of temperature activity profiles and thermostability

Thermal properties of inulinase were determined in the standard assay mixture. To determine the optimum temperature, assays were carried out in the range of 40–60 °C. Thermostability was examined by incubating the enzyme, added to acetate buffer (pH 5.0), for 1 h at different temperatures (30–75 °C) prior to the inulinase assay.

### Column hydrolysis

Continuous hydrolysis of inulin was carried out using a low-pressure chromatography system consisting of a glass chromatography column (1 × 20 cm, Bio-Rad Labs., Hercules, CA, USA), a peristaltic pump P-50 and a Frac-100 fraction collector (both from Pharmacia, Uppsala, Sweden). 3 g (wet mass) of inulinase immobilized on BES.DM-GMA (1:1) polymer (about 600 U) was packed, under gravity, in a column equilibrated with 0.02 M acetate buffer (pH 5.0). A solution of inulin (0.5 % in 0.02 M acetate buffer, pH 5.0), was pumped through the column at various flow-rates (0.1–4.0 mL/min) in a first experiment, and at a constant flow rate of 0.5 mL/min during a 28-day-long continuous hydrolysis experiment. To prevent growth of microorganisms, sodium azide (0.01 %) was added to the inulin solutions. Samples of the column effluent were taken at regular intervals, and the reducing power was monitored. Continuous hydrolysis experiments were conducted at room temperature, because the column did not have a thermoregulatory system.

The efficiency of inulin hydrolysis was determined by comparing the reducing properties of the products (calculated as fructose equivalent) with the theoretical hexose amount obtained after complete hydrolysis of inulin:

$${\text{Hydrolysis}}\;{\text{efficiency (}}\% )= \frac{{{\text{fructose}}\;{\text{equivalent}}\;{\text{in}}\;{\text{the}}\;{\text{hydrolysate}}\; ( {\text{mg)}}}}{{{\text{amount}}\;{\text{of}}\;{\text{hexose}}\;{\text{after}}\;{\text{complete}}\;{\text{hydrolysis}}\, ( {\text{mg)}}}} \times 1 0 0\;\%$$

### Analytical methods

Carbohydrates were analyzed by HPLC (Shimadzu, PROMINENCE LC-20A) using a REZEX RSO Oligosaccharide column (200 mm × 10 mm, Phenomenex) coupled to a refractive index (RI) detector. Elution was performed with Milli-Q water (40 °C) at 0.25 mL/min. The total inulo-oligosaccharides were estimated as the sum of inulobiose (*F*_2_) and other oligofructosides (mainly Fn and trace amounts of GFn) with degrees of polymerization (DP) ranging from 2 to 6. The degree of polymerization of the products was identified by comparing the elution times of the mono- and oligosaccharides with those of appropriate standards. Fructose, glucose, sucrose, kestose (DP3, GF_2_) and nystose (DP4, GF_3_) were used. The results were confirmed by determining the molecular weights of the products by flow injection HPLC-ESI/MS using an Agilent Technologies LC-QQQ 6460 with a quadrupole ion trap mass analyser. Mass spectra were recorded in negative and positive-ion electrospray modes in the range of 200–1600 m/z.

## Results and discussion

**Fig. 2:**
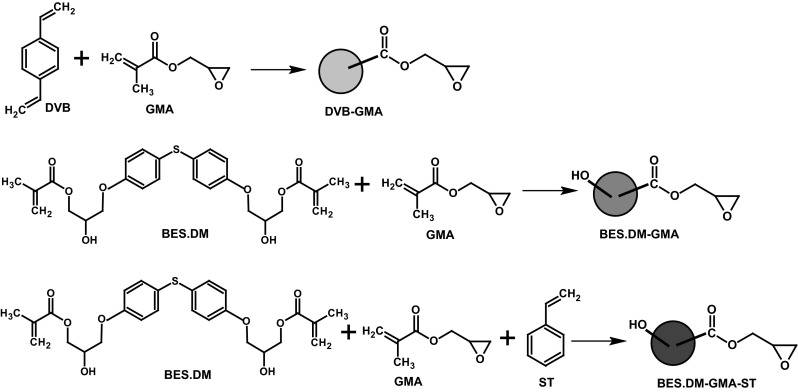
Synthesis and the chemical structure of the monomers used for their copolymerization

The aim of the present work was to develop an efficient procedure for the immobilization of crude (non-purified) inulinase for continuous hydrolysis of inulin, using new types of polymers containing epoxy groups. For this purpose, four types of glycidyl methacrylate copolymers in the form of microspheres were synthesized by suspension–emulsion polymerization. The process was performed at variable concentrations of the functional monomer (GMA), which is why different numbers of epoxy groups were obtained, ranging from 0.29 to 2.79 mmol/g (Table 1). The crosslinking agents were *bis*[4(2-hydroxy-3-methacryloyloxypropoxy)phenyl]sulfide and divinylbenzene. Crosslinked polymers had a high chemical and physical resistance, which is necessary for their use in biological systems. The chemical structures of the studied monomers and microspheres are shown in Fig. 2.

**Fig. 3:**
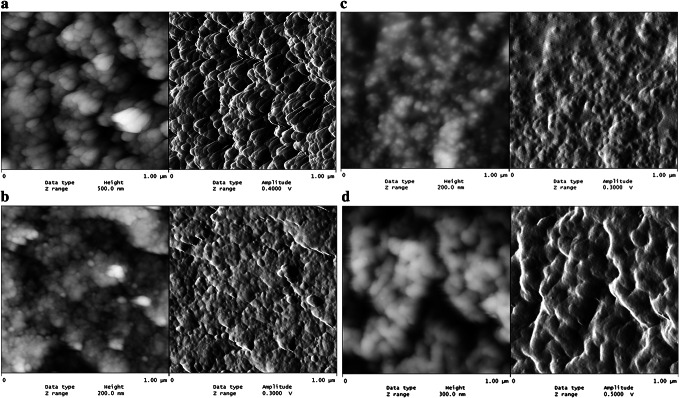
Contact-mode (AFM) images of microsphere surfaces **a** DVB-GMA (1:1), **b** BES.DM:GMA (1:1), **c** BES.DM:GMA (1:6), **d** BES.DM-GMA-ST. Magnification of all photos was ×135,000

FTIR data for the copolymers are listed in Table 2. In the FTIR spectra of the copolymers studied, C-H stretching vibrations of methylene and methyl groups of the aromatic aromatic ring backbone were observed at 3049–3059 and 2925–2932 cm^−1^, respectively [29]. The epoxide group gave a shape signal at 905–908 cm^−1^. Aromatic skeletal absorption was observed at about 1591–1604 cm^−1^. The signal of a C=O group occurred at approximately 1728–1730 cm^−1^. The images of the surface texture of the microspheres DVB-GMA (1:1), BES.DM-GMA (1:1), BES.DM-GMA (1:6) and BES.DM-GMA-ST (1:1:3) show that the copolymers obtained in the study had a varied structure (Fig. 3).

**Table 2:**
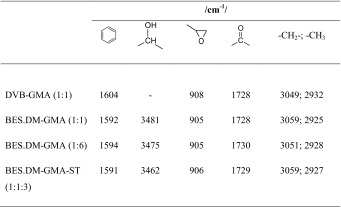
FTIR data

Table 3 shows characterization of the porous structure of the microspheres obtained by the nitrogen adsorption–desorption method. The largest specific areas and pore volumes were observed for the copolymers DVB-GMA (1:1) and BES.DM-GMA (1:1), while the copolymers BES.DM-GMA (1:6) and BES.DM-GMA-ST (1:1:3) were rather non-porous. This indicates that porous structure is preferentially formed in the molar ratio of monomers 1:1. The larger the share of a difunctional monomer (GMA and ST), the lower the porosity of the obtained polymer.

**Table 3:** Porous structure of the obtained microspheres

Copolymer	Specific surface area (m^2^/g)	Pore volume (cm^2^/g)	Average pore diameter (Å)
DVB-GMA (1:1)	87	0.44	200
BES.DM-GMA (1:1)	80	0.44	220
BES.DM-GMA (1:6)	5	0.01	–
BES.DM-GMA-ST (1:1:3)	23	0.12	105

**Fig. 4:**
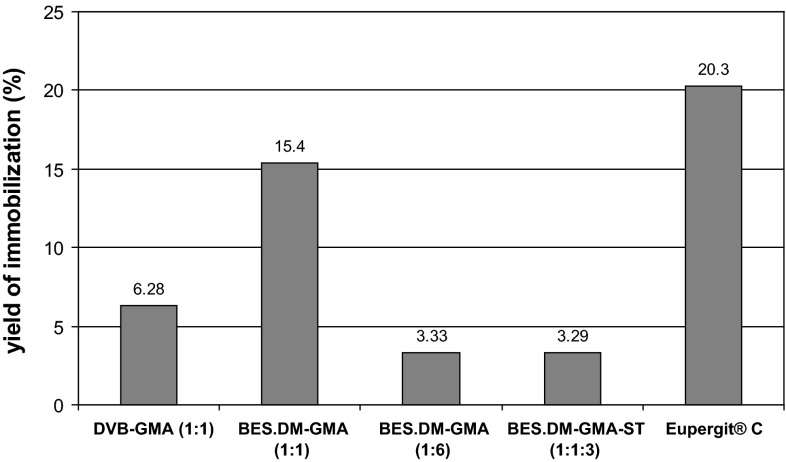
Immobilization yield of crude inulinase on the newly synthesized polymer microspheres and the commercial carrier, Eupergit^®^. Conditions: 1 M phosphate buffer, pH = 7

In a further study, carried out under optimal conditions for Eupergit^®^ C (data not shown), we examined the ability of the four carriers to immobilize inulinase from crude post-culture medium (Fig. 4). A decision was made to use crude inulinase (as a concentrated medium obtained from a culture of *A. niger*) instead of the purified enzyme, because the latter, being an endoinulinase, did not hydrolyze sucrose. When the culture supernatant was used, fructose was produced from inulin, suggesting that apart from endoinulinase, an exo-acting enzyme was also present in the culture filtrate [38]. The highest efficiency of inulinase immobilization (15.4 %) was achieved for BES.DM-GMA (1:1) and the lowest for the carriers BES.DM-GMA (1:6) (3.29 %) and BES.DM-GMA-ST (1:1:3) (3.33 %). This result correlates with differences in the porosity of the polymers and may suggest that the yield of immobilization depends on their specific surface area. Ettalibi and Baratti [6] immobilized inulinase from *Aspergillus ficuum* on porous glassware activated with 3-aminopropyltriethoxysilane in toluene reaching an efficiency of inulinase immobilization varying from 29 % (3.000 Å) to 71 % (80 Å), depending on pore diameter. A high immobilization yield (82.6 % of inulinase specific activity) was also obtained by Paula et al. [28], in a gelatin–water support after treatment with glutaraldehyde as a cross-linking reagent. The authors did not report the performance of the obtained biocatalyst in semi-continuous conditions, though. In another study, partially purified exoinulinase was immobilized onto Amino-cellulofine using glutaraldehyde as the cross-linking agent, with an immobilization yield of 15 % based on the enzyme activity [13].

In this present study, BES.DM-GMA (1:1) was used for all further experiments; the yield of enzyme immobilization on this polymer was as high as 76 % relative to inulinase immobilization on Eupergit^®^ C under optimal conditions.

Factors affecting inulinase immobilization were studied using phosphate buffers of different pH and molarity. The range of pH chosen for the investigation coincided with the pH range for the enzyme in solution. Data presented in Table 4 show that the highest efficiency of enzyme immobilization was obtained at pH 6.5 (19.73 %) and the lowest at pH 8 (10.57 %). These data are close to those of Nakamura et al. [23], who obtained the highest immobilization efficiency in 0.1 M acetate buffer at pH 6.0 for inulinase immobilized on Amino-Cellulofine. Lower pH values of pH 5.0, pH 5.5 and pH 3.5 have been reported by Wenling et al. [43], Singh et al. [35] and Paula et al. [28], respectively. These differences can be caused by the specificity of our enzyme and the carrier used for immobilization. We also tested Sorensen buffer at pH 5.0, 5.5 and 6.0, but the effectiveness of immobilization using this buffer was lower than for phosphate buffer: 14.06, 14.73 and 14.19 %, respectively (data not shown). Interestingly, when the molarity of the phosphate buffer was decreased to 0.75M, the yield of enzyme immobilization (19.44 %) on our polymer surpassed that obtained on Eupergit^®^ C (18.94 %) under optimal conditions of immobilization.

**Table 4:** Some factors affecting the efficiency of inulinase immobilization on a BES.DM-GMA (1:1) polymer microsphere

Factor varied	Efficiency of inulinase immobilization (%)
Phosphate buffer pH (1 M)	
6.0	16.26
6.5	19.73
7.0	17.53
7.5	12.82
8.0	10.57
Concentration of phosphate buffer pH 6.5 (M)	
1.0	15.60
0.75	19.44
0.5	18.30
0.25	17.25
Eupergit^®^ C (1 M, pH = 7)	18.94
Amount of carriers	
0.1	10.41
0.2	14.91
0.3	15.07
0.5	14.83
1.0	15.64

In a subsequent experiment, the influence of the amount of the carrier on the efficiency of enzyme immobilization was determined. In the range of 0.2–1.0 g of the carrier, immobilization efficiencies were similar; a slightly higher efficiency was obtained for 1 g of the carrier. For economic reasons, 0.2 g of the carrier was used in further experiments (Table 4).

**Fig. 5:**
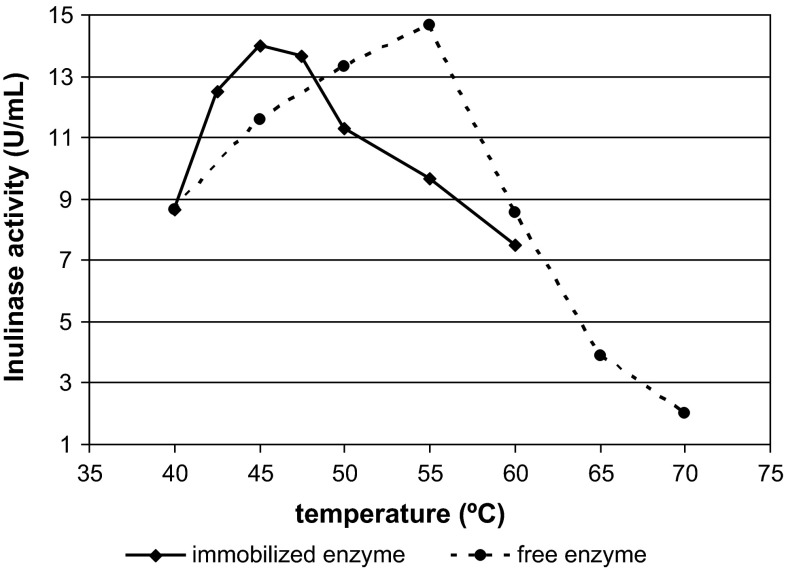
Activity of free (**a**) and immobilized (**b**) inulinase from *A. niger* 20 OSM at different temperatures

The optimal temperatures for free and immobilized inulinase were 55 and 45 °C, respectively. A further increase in the temperature, significantly reduced the activity of the enzyme (Fig. 5). At 50 °C, the activity of immobilized inulinase decreased by 20 %. As far as we know, there are no reports of a similar downward shift in optimum temperature for immobilized inulinase relative to the free enzyme. Generally, the optimum temperatures for immobilized inulinases are higher than those obtained for their free counterparts [6, 28, 35, 43]. However, there are some reports concerning other hydrolases that resemble our results, e.g., cellulases immobilized on Aminosilica-1 [14], beta-fructofuranosidase immobilized on FE-4611 carrier resin [21] and lipase immobilized on porous chitosan polyphosphate beads [18].

**Fig. 6:**
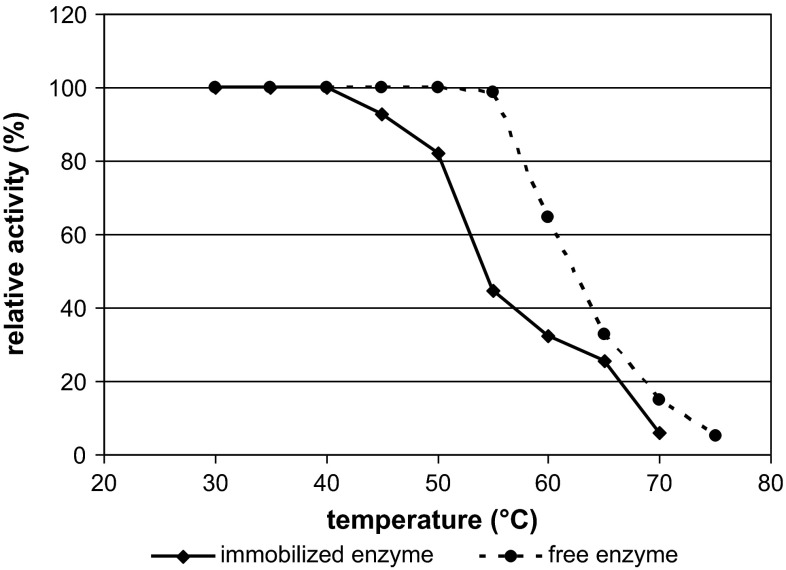
Thermostability of immobilized inulinase on BES.DM-GMA (1:1) polymer microspheres

In the present study, immobilized inulinase was thermostable at 40 °C for 60 min; however, when the temperature was increased to 55 °C, its stability significantly decreased to 44.73 %. At 65 and 70 °C, the enzyme reached only 25.57 and 5.81 % of its initial activity, respectively (Fig. 6). At 50 °C, inulinase retained 82 % of its initial activity (for 60 min). These values are quite different from those reported for immobilized inulinases coming from some other microbes: *F. oxysporum* (50 % activity at 50 °C after 45 min), *A. niger* (stable for 30 min at 60 °C) and *A. candidus* (stable for 60 min at 55 °C) [9, 15, 23]. The thermal stability of immobilized inulinase from *A. fumigatus* at 60 °C was considerably higher (~70 % up to 48 h). Mazutti et al. [20] reported half-lives of 2224.0 and 322.0 min at 50 and 55 °C, respectively, and Treichel et al. [41] found half-lives of 4158.0 and 594.0 min at 50 and 52.5 °C, respectively, for free inulinase from *K. marxianus* NRRL Y-7571.

**Fig. 7:**
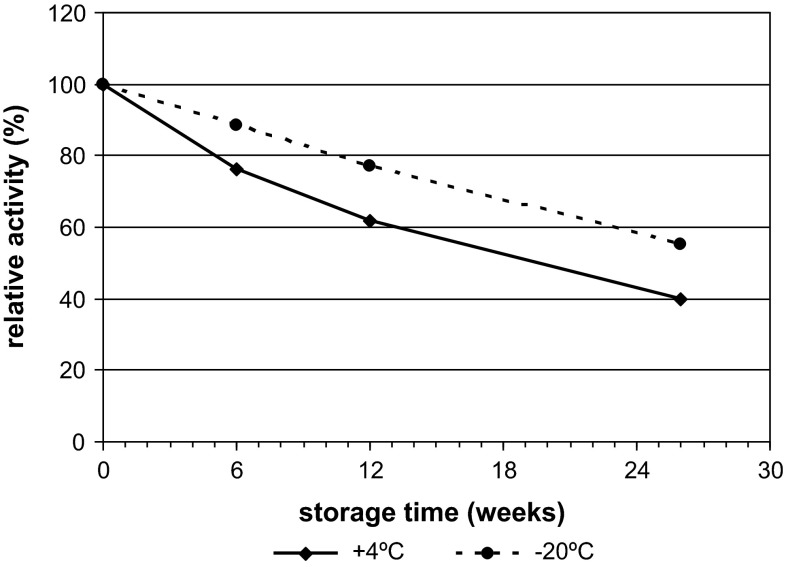
Influence of storage temperature on the activity of immobilized inulinase

Our study of long-term storage (for 182 days) of immobilized inulinase at 4 and −20 °C showed that the enzyme lost 60.1 and 44.9 % of its activity, respectively (Fig. 7). Inulinase immobilized on gelatin by Paula et al. [27], lost only 9.8 % of its activity when kept in a refrigerator (+4 °C) for 34 days, whereas its free counterpart, stored in the same conditions, lost as much as 22.5 % of its activity. Inulinase immobilized on BES.DM-GMA (1:1) kept at 4 °C lost about 24 % of its activity after 42 days; in turn, the enzyme kept at −20 °C retained almost 89 % of its activity. These differences may result from the different ways in which the enzyme was protected against dryness and activity loss.

**Fig. 8:**
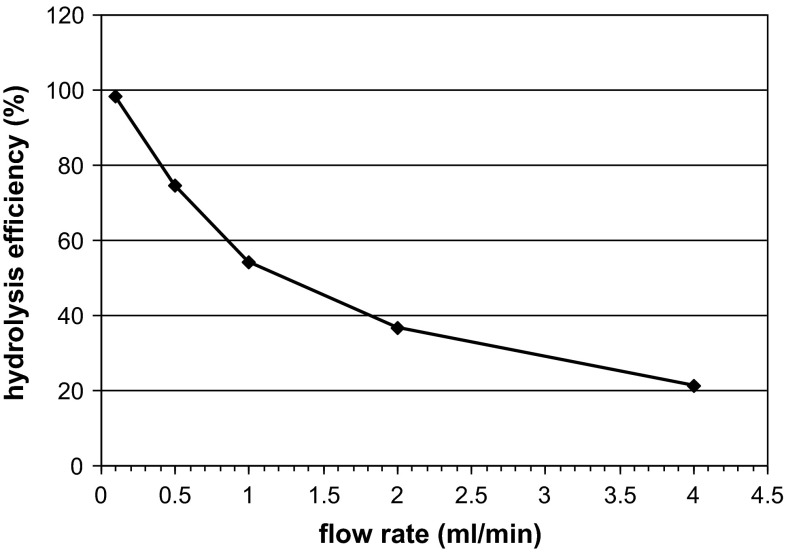
Effect of flow rate on efficiency of inulin (0.5 %) hydrolysis by *A. niger* 20 OSM inulinase immobilized on BES.DM-GMA (1:1) polymer

**Fig. 9:**
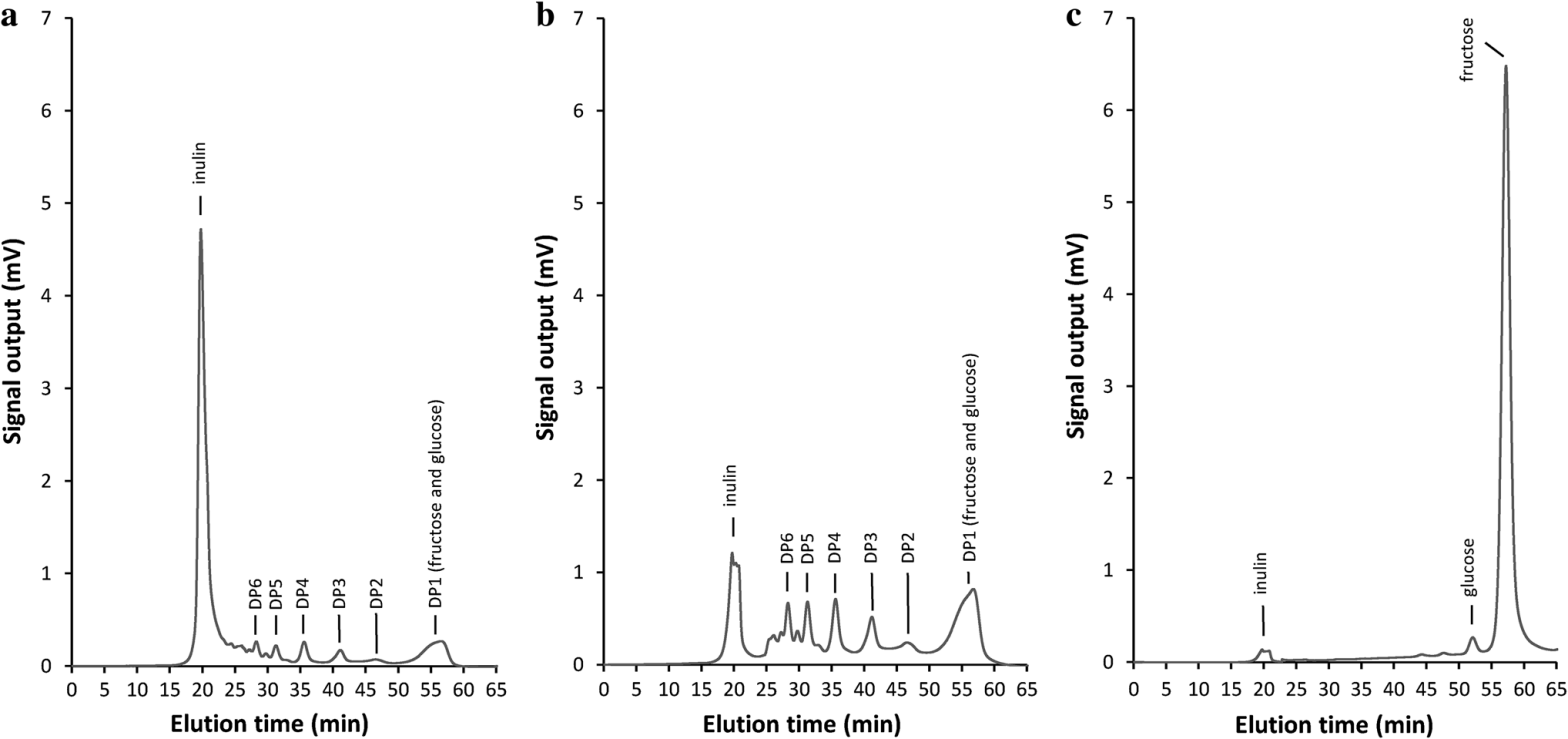
HPLC-RID chromatograms of the products of inulin hydrolysis in continuous conditions using *A. niger* 20 OSM inulinase immobilized on BES.DM-GMA (1:1) polymer in a packed column at a flow rate of 4 mL/min (**a**), 1 mL/min (**b**) and 0.1 mL/min (**c**)

**Fig. 10:**
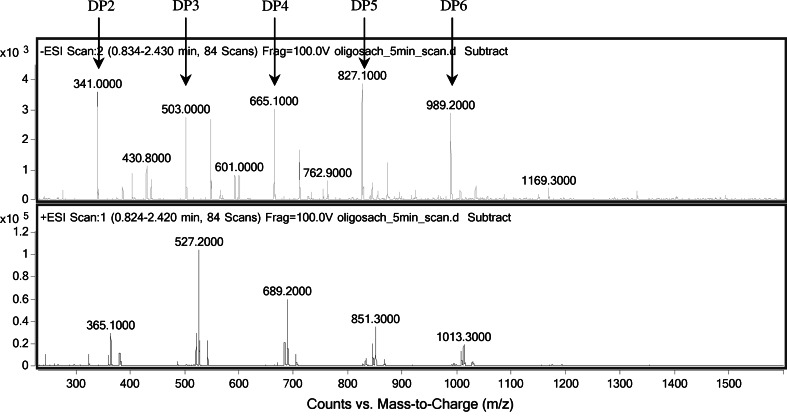
LC-ESI/MS spectra of the products of continuous hydrolysis of inulin (at a flow rate of 1 mL/min) by *A. niger* 20 OSM inulinase immobilized on BES.DM-GMA (1:1) polymer on a packed column

As the last stage of the present experiments, we tested the ability of the immobilized enzyme to work in continuous conditions in a chromatographic column. The effect of flow rate on the efficiency of inulin hydrolysis by immobilized inulinase from *A. niger* 20 OSM was examined. Figure 8 shows hydrolysis efficiency as a function of flow rate through a reactor column packed with immobilized inulinase. An increase in the speed of flow from 0.5 to 4 mL/min resulted in an about 3.5-fold decrease in the efficiency of inulin hydrolysis, which could have been caused by too short a contact of the enzyme with the substrate. The results obtained by other authors have shown that the effect of the flow rate of the substrate on hydrolysis yield depends on substrate concentration. In a packed-bed reactor containing 412 U inulinase, dahlia inulin at a concentration of 7.5 %(w/v) was completely hydrolyzed at a flow rate of 2.0 mL/min at 60 °C, whereas for a medium with 2.5 % inulin at 60 °C, the flow rate was slower (1.0 mL/min). The reactor was successfully operated over 30 days without loss of inulinase activity [13]. A detailed analysis of the hydrolysis products by HPLC-RID and LC-ESI/MS techniques (Figs. 9, 10) revealed that in our system, at a flow rate of 0.1 mL/min, inulin [at a concentration of 0.5 % (w/v)] was almost completely hydrolyzed to fructose with only residual amounts of oligosaccharides detected. The total concentration of oligosaccharides increased at higher flow rates, with the oligosaccharide (DP from 2 to 6) yield in the overall hydrolizate solution rising up to ~41 % at 1 ml/min, as shown in HPLC-RID chromatograms (Fig. 9). At this flow rate, the relative amounts of the respective oligomers were estimated as follows: DP2 (3.8 %), DP3 (7.8 %), DP4 (10.6 %), DP5 (10.2 %), DP6 (8.4 %). The occurrence of both fructose and oligosaccharides in the hydrolysate suggests that two forms of inulinase, an exo- and an endo-acting enzyme, were immobilized on our carrier.

**Fig. 11:**
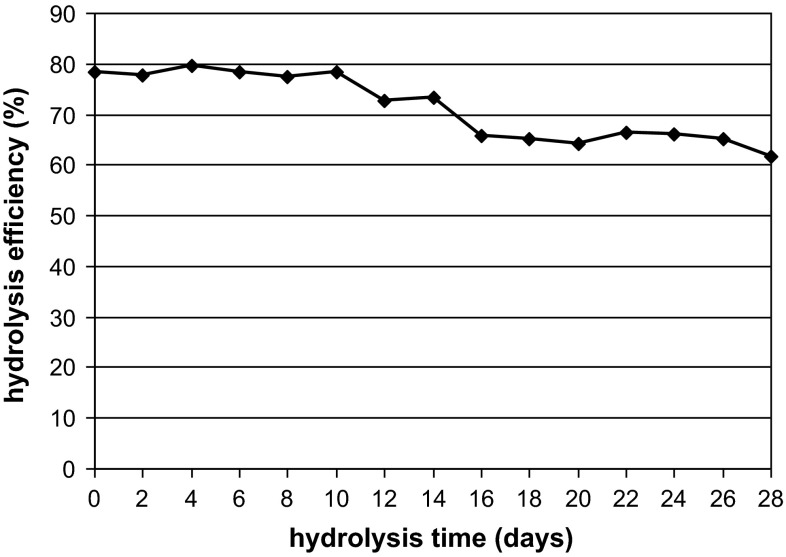
Operational stability of a packed column in continuous hydrolysis of inulin using *A. niger* 20 OSM inulinase immobilized on BES.DM-GMA (1:1) polymer

The operational stability of a packed column in continuous hydrolysis of inulin by inulinase from *A. niger* 20 OSM immobilized on BES.DM1-GMA1 is shown in Fig. 11. During the 28 days of operation, only a slight decrease in the efficiency of inulin hydrolysis was observed. After this time, the efficiency of inulin hydrolysis dropped by only about 20 % in comparison to the initial value. This is a promising result from the practical point of view. Our results are similar to those of Kim et al. [13], Nakamura et al. [23] and Yun et al. [45]. In their experiments, columns containing inulinases from *A. niger* or *S. cerevisiae* immobilized on Amino-cellulofine worked continuously for 45 and 30 days, respectively, without a significant loss of inulinase activity. Less favorable results were obtained for inulinase from *Kluyveromyces* sp. Y-85 immobilized on macroporous ionic polystyrene beads [43], where only 49 % of the initial activity was retained after 35 days of continuous hydrolysis. A sol–gel immobilized inulinase used by Santa et al. [33], for the hydrolysis of inulin to fructose displayed a promising operational stability, since it was used in more than 20 consecutive 24-h batch runs without a noticeable decline in product yield. The kinetic parameters estimated from the typical Michaelis–Menten kinetics suggest that immobilization in sol–gel did not tamper with the native enzyme conformation, whereas entrapment brought along mass transfer limitations.

Enzyme immobilization alters some properties of enzyme molecules, such as their catalytic or thermal stability, in comparison to their soluble counterparts [6, 28, 35, 43]. This change of properties may be due to either changes in the intrinsic activity of the immobilized enzyme or due to the fact that the interaction between the immobilized enzyme and the substrates takes place in a microenvironment which is different from the bulk solution. Changes in catalytic properties upon immobilization may also be due to the changes in the three-dimensional conformation of the protein as a consequence of the binding of the enzyme to the matrix.

Our study shows that the number of epoxy groups may be a crucial factor both for the efficiency of covalent binding of an enzyme and its activity. Eupergit^®^ C is known to have a number of epoxy groups (0.6 mmol per dry g of polymer), due to which it has the ability to immobilize enzymes directly by covalent attachment [11, 12]. From our results it seems that an enzyme may attach directly to a polymer via too many bonds with epoxide groups. This is borne out by the finding that the activity of inulinase on BES.DM-GMA (1:6) (with the highest distribution of epoxy groups, 2.79 mmol/g) was significantly decreased in comparison with BES.DM-GMA (1:1) (1.4 mmol/g) (Table 1). Then, the protein is likely to exist in a close proximity to the polymer surface, which may change the spatial structure and, thus, the activity of the enzyme. Generally, the operational stability of an enzyme is improved on immobilization, which is why the concept of stabilization has been an important driving force for immobilizing enzymes [28, 42].

Studies carried out by numerous authors using different methods have established a relation between stabilization and the number of covalent bonds to the matrix [16]. One of the main problems linked with the use of immobilized enzymes is the loss of their catalytic activity, especially when the enzymes are acting on macromolecular substrates [8, 17]. Because of limited access of the substrate to the active site of the enzyme, the enzyme activity may be reduced to accessible surface groups of the substrate only. This steric restriction may, in turn, change the characteristic pattern of products derived from the macromolecular substrate [2]. There are several strategies to avoid the steric problems, such as selection of supports composed of networks of isolated macromolecular chains and careful choice of the enzyme residues employed in immobilization techniques along with the supports used for enzymes [28].

## Conclusions

Estimates of the costs of obtaining the new polymers show that they can be cheaper than the very expensive commercially available carriers of this type (e.g., Eupergit^®^ C). The polymers synthesized in our study are stable at room temperature, as opposed to Eupergit, which requires a low storage temperature (−20 °C). The significance of the data presented here is also that the immobilization of non-purified inulinase from *A. niger* using the new types of polymers is a simple method that gives good operational stability of the enzyme on a packed column in continuous hydrolysis of inulin (leading to complete hydrolysis to fructose or to oligosaccharides of a high commercial value). The present results promise future improvement of inulinase immobilization, which can be achieved by optimizing polymer porosity, the specific area of polymer microspheres and the number of epoxy groups on the polymer surface as well as by performing immobilization processes by an indirect method using a spacer to enhance enzyme mobility. Development of multifunctional terpolymers by using monomers with different functional groups (to introduce hydroxyl, amide and epoxide functions directly into the surface) should also be attempted. Experiments with various ratios of epoxy to amide groups should be accompanied by an investigation of the conditions of inulinase immobilization, which may also improve the performance of this biocatalyst.
